# Supplementary material for: Tracing the function expansion for a primordial protein fold in the era of fold-based function prediction: β-trefoil
Source: PLoS One. 2025 Jul 3;20(7):e0320177. doi: 10.1371/journal.pone.0320177 (PMC12225799; doi:10.1371/journal.pone.0320177)
Supplement: S2 File — (PDF) [file pone.0320177.s002.pdf]

```
>7KDU_chainB/1-66
--NGLCVDVRDAIQLWPLWTLKRDNTIRSNGKCLTTYVMITRWQIWDNGTIINPRSSLVLAATTTLTV
>3AH2_chainA/1-67
NNQSDRFLSKNIVL-WQKWIIEYNYTLKCQENYLTWYVETSYWNINYLDNLYNLQDTNVLDVYTHVIV
>4HR6_chainC/1-68
NEDALCVDVAGRLILYPKWTFHSDGTVRSLGKCLATLVVIKSWDVSVGGTIMNNYEDLALTSNTNLTM
>5G56_chainA/1-66
TIAGKTLQEVTSIITADHWKIQHIGRISSAGRGNWN-GTVTCFIIGDGYYRIVVGDGTNLQISK-IEG
>5TPB_chainA/1-65
LKPNKYV--DVYMYLKG-FIIKKYASNVVVKNKELAILSADVVVMKSKNKMNLQDNNGFIGFHAKLVA
>1HWM_chainB/1-68
ETDGLCVDVRNPIQLWPQWTFYNDKTIRSMGKCMTAYIMITKWEVLIDGSIINPSSGLVMTAPTTLLL
>3ZXD_chainB/1-66
RQEKEYMTVVSAATLGHFVLYEDWGRIKTLNTMYAYGIYF-RWALRHGDVVTKYFTRSGLCYD-NVYC
>1ba7/1-68
DFIFGGIRAAPPLVQSRGTIISHPLSLKFDPTEWSVAVKIDWFRLERNYKLVFCPKCGDIGISRRLVV
>1N4K_chainA/1-68
GGEQKFLTCDEHVFLATLWEVEVVFRFKHLATYLAASLVSEIFELDPYVRLRHLCTNTWVHSTLKIGT
>4GOV_chainA/1-64
FAVTRYAHLSPEIAVDRLITLAFQDQQTADHRFL--RHDG-GYLEFRSGKVFRDCEGRYLAPS-TLKA
>5Y5W_chainA/1-29
IS---------VEHMF--------------EWRGMVYITYV----------------MYQLLDDLRIM
>5FV9_chainE/1-66
DHQGTNCLDTLV-GVYEEWALTKEKSVKHMDLCLTVLIKLEKWEQEGNSKLRHVGSNLCLDSRG-LSV
>2vsa/1-68
NNKNIVVTLDSPVESYKKWNIKYAYKIYNRELLLSWVIRGGYWTIEKNYKFRNLSDSKILDLKTPLVV
>4CJM_chainA/1-67
QLTSGHIQVLG-ISARGQLLVGSQVRIKGTEFYLCMKLVGDVFIEKVLEALMSAKYSWYVGFTGRPRK
>4owj_1/1-68
MASDLCLDVYGSVNGWSVWGLDKEERYRSSDRCLTVTLTVCKWYWEGDKLISRYVDGYLLNIVRNVQV
>8ae4/1-65
ASTGEYATVEGPVKAEPIWQVTRQYTIKYQGYGFSYPVIA-EYILTADVYIIRGVDVEVGV--NTLVY
>6lf1/1-65
VTEGGRAFYNLDLGIF-IWSFQKSDTIGRESKFLQY--IMQLWSLEEFYRLLNKVHKAYLDYNGDLVA
>5uc6_2/1-68
NVALNQSIIRAYLTAAAKFDMGAYVILRISKTLYVTPVLLGLFFWETHGYFTSVAHPLFIATKYWVCL
>2hth/1-43
RFYDAGTLLLSRLIWRD---------------AILL-----FIEEQAAGKIVVHLHPSYIKLS-----
>3VSZ_chainA/1-68
IPNGKVLDVLDQIVQWTQWYLGGGYKKIVSGRALDVVLIQGHWKFTGYYKISSRHCGKLIDVWGIIQQ
>3PHZ_chainA/1-67
SFYNTRLALSEDVII-SLWLIEPTYTVRNAFSYMDLAIIGPKWIISQIWKIKSKETGTFVTLLGTVVG
>5bqu/1-68
DYLNKVVQQVDNVNLYTKWTIRYNYQFFNTILVLTWTVRVDYWLINPVYTITNLRDTKALDLYTAIQV
>2IHO_chainA/1-68
SLVPSAIDLKDPIVGWFLWLAEPTFTLCNLFTYMDLAVNGALWTIKTSYKIQNYGSKTFVDLGAKIAG
>1pum/1-68
DDNGMTVDVRDQIQLWPLWTIKKDGTIRSNGSCLTTYVMITIWQIWGNGTIINPRSNLVLAASTTLTV
>6ifa_1/1-68
EAEGNVIDIQGNAIIYPLFFIDQIGWIISVRKALTVDIVQYQWIFEDNPIIRCYENPLVLSVTDKVCL
```

>3wmu/1-67
GSAGKFLHPKGNLVLHSYFQFDRWGYIKHAGKIVHPKLVLHLFAMDFFDNIIHKAG-KYVHPKTLTVM
>7zoh/1-66
GSNSKYVNSRNAMWCDSLFTVIDAGNLRGNNGLYSS-MTCIVFDWIGSVSLRGS-NGMYVSSEQAITC
>1IJT_chainA/1-67
GIVGFHLQALPRIGGAHLLLSPVERGVVASRFFVA-KLYGTTFKEILLNAYESYKYGMFIALGKTKKG
>1PWA_chainA/1-62
PIGSCFLRIRAVVDCARLLEIKAVALRVHSVRYLC-KMQGSAFEEEIYNVYRSEKHRLPVSLS-----
>3F1R_chainA/1-68
RRTGFHLQILPSVQGTRILEVAVGLVSIDSGLYLGMELYGEIFREQFEETYSSNIYKYFVALNTPRDG
>1a8d/1-53
YD--------------SKFIIKRFIKLYVSYHIVGYILRVGKMEAVKSVQLKLYDSLGLVGTHILI-A
>8ae5/1-67
GFAGMYASSKDPVTA-ERWWIARDYTITEFRIQWSRPVYLKAWRIQPVYHIVGNVSTDWADLRPQVYM
>5EC5_chainA/1-66
IIEKEYMTVVSAATLGHFVLYEDWGRIKTLNTMYAYGIYF-RWALRHGDVVTKYFTRSGLCYD-NVYC
>2go2/1-68
SSVGHAGLALAVVLDPHPVRFESFLNIKFGPGVWDVAVKVTPFKVEKEGKIVYYPERLDIGLVYYLAV
>8hnr/1-68
KEFRGGLTLAKLNVQAELPAMIWYLNIEFQPLSWKVEVKIESFKIEPYRKLVYCESCKDLGISRLLVV
>2gzb/1-67
HMVGEGGLALAV-VLDPLTVRFETLNIKFVPSEVWDAVKVDPFRVEKEGEIVYYPDRGDIGLVYYLAA
>4zot/1-68
SSSHHAGLALAAVVLDPLPVRFESLNIKFGPSVWDVAVKVSFKVEKEGEKIVYYPERLDIGLVYYLAV
>1r8n/1-66
SDIIGGGVRPGII--QELPVRFSSLEIEFVEKKWVIRVAIEFFKIEKLYKLVFCPKCSDIGINRSLVL
>3tc2/1-68
PSTFWDVYLGKGVFRYNTPVRFILLNIQFAITIWKVLLETQWFKIVKSYNLLYCPVCLKVGVVRRLAL
>5fnx/1-68
PLIGRDVYLGKGVFRYNTPVRFILLNIQFNITIWKVLLETGYFKIVKGYNLLSCPFCAKVGVVRRLAL
>6MU1_chainA/1-68
DIEQEKFLTCDHVFLRTLWEVESLFRFKHLGHYLAASLVSGIFELDPTVRLRHLCTNTWVHSTLKIGT
>1xzz/1-49
SFLNKYLTVNKRVTLDEWFYIQDKVVLNPVGQPLHAEVNST-KIVLFLEH------------------
>3uj0_2/1-68
DNEQEKFLTADHVFLRTLWEVESLFRFKHLGHYLAASLVSEIFELDPVPRLRHLATNTWVHSTLKIGT
>3llp/1-67
SCNERNVSTRQDLSANQTFQEIDKCAFRHTGKYWTLGVQSKYFDIEWRITLRASNG-KFVTSKGQLAA
>5A1H_chainA/1-37
TH----------------------------GKAVEHEWR--MVLAQAPWFYITYEKVLYMYQLGDLR-
>4UY4_chainA/1-28
--LKAVEHVDEWKGMVLFYITYEKDPVLYM
>1ira_1/1-68
SSVNQKTFYLRQLVAGYEKIDVVPFLGIHGGKMCLSLQLETAFIRSDTTSFESAAPGWFLCTAQPVSL
>1i1b_1/1-67
VRQ-QKSLVMSELKALHVVFSMSFVALGLKEKLYLSLQLEKVFNKIEKLEFESAQPNWYISTSMPVFL
>5bow_1/1-68
SSYQKALYTRDQLLVGDKICTLPNRFLGIQGGSCLALQLEETFFQSSSFRLEAAAWGWFLCGPQPVQL
>5duy/1-67
MTAGKFLHPYGKLVLHSYFQFDRWGYIKHVGKIVHPNMVLHLFAMDFFDNIMHKGG-KYIHPKTETVI

```
>4ouj/1-68
NFLGKVVQQVSAVNLYIKWTIIYNYQFFNKILVLTWTVRVAYWLINPVSTITNLRDKKVLDLYTTIQV
>AF-A0A3D9WMQ9-F1-model_v4/1-67
SFENGCLTTFY-TSLGDKWTFRGSVKVVNVGSCLSAAVFVLLWKTGSGNTLKAVYGGGCLDLADGAEE
>AF-A0A191US67-F1-model_v4/1-66
--HGKVLAVDGRVVQFDAWQLGDGWYLIRSGKVLGVQVVQGLWALVGWYRVRNRHSGKVLAVMARVVQ
>AF-A0A1I6E245-F1-model_v4/1-67
VE-ALCLDNNAPVQIWGKWRVVADGTIAISGKCLDIAVQLGQWRVRSDGSVQNPQSGLCLDNATRLNI
>AF-A0A820M8C8-F1-model_v4/1-66
KMAHKVLDSNSDVYTLDKVFIQQDDGNAATSFVL--HMYTGKWRFHRSYVMKNLATSRVLDSNGNAYP
>AF-A0A4Q2KX23-F1-model_v4/1-68
AVGSGCIDAVDLVQQWAQWTLTDSYRITNVGLVLQVPLELKQFELVGFYQLVNQHSGLCAAVGAAIAQ
>AF-A0A3L7BAS7-F1-model_v4/1-67
RIGGKCLDVDNKVQLWTSWS-RVGDTYRALGKCLDVKVQLGVWQPQSDGSIRNPQSGKVLEAATQIQI
>AF-A0A318NTU0-F1-model_v4/1-67
TT-GRCVDVPGQVSLWDRWTYTTGKALTVGNKCLDAQVTIGQWNVNANGTITGVQSGLCLDPQTKLIL
>AF-A0A844M7R0-F1-model_v4/1-68
VRESKQYLVNVWPQLSNELKIDGGEIKTTEQAVTLGNCYYGGWRITKASGITNISYHNRLALDGYITT
>AF-A0A3A9W2R1-F1-model_v4/1-68
GRKGLHMDVANNIQQWGQWQVGNGYYRLKSGKSITVNIESGEWQIIGYYRLKSRDSGKSIGVGANVES
>AF-A0A820T4Y8-F1-model_v4/1-68
SGEQKFLTCDNVVFLATLWEIEVVFRFKHLATYLAAALIPHLFELDATTRLQHICTNTWVHSTFKIGC
>AF-A0A6I5GSK5-F1-model_v4/1-68
AAAGKCLDVAGAVQIYDQWTVGADGTLRALGKCLDVKVQLGKWTVTAAHDIVNPQADKCLDVNSRAQI
>AF-A0A0U3MKC8-F1-model_v4/1-68
ALLSLCLDLANGVVQAARFDIADGSYRLRSGKVLDVVLQQDHFAALAAIQLKAQHSGLCLDLEAPIQQ
>AF-R8CKC2-F1-model_v4/1-68
MDKQKYMDVDSWLDQYEKWIIDGGYYVLISGRMVDVMLNQNQWNTEGYIAFQNKNSGKYADVNDHLNQ
```
