# Supplementary material for: Tracing the function expansion for a primordial protein fold in the era of fold-based function prediction: β-trefoil
Source: PLoS One. 2025 Jul 3;20(7):e0320177. doi: 10.1371/journal.pone.0320177 (PMC12225799; doi:10.1371/journal.pone.0320177)
Supplement: S3 File — (PDF) [file pone.0320177.s003.pdf]

```bash
#!/bin/bash

# Exit on error
set -e

# === FIGURE 7a: Identify β-trefoil fold containing proteins using DALI ===

echo "Downloading representative β-trefoil structure from PDB..."
wget -nc https://files.rcsb.org/download/5bow.pdb

echo "Please upload 5bow.pdb manually to the DALI server at:"
echo "https://ekhidna2.biocenter.helsinki.fi/dali"
echo "After downloading results, proceed to filter output with the following command:"
echo

# Example: Filtering DALI output for RMSD < 2.0 Å and coverage ≥ 80% (assuming query length = 151)
QUERY_LENGTH=151
awk -v qlen=$QUERY_LENGTH 'NF > 0 && $1 !~ /^#/ && $1 !~ /^Job:/ && $1 !~ /^Query:/ && $4 / qlen * 100
>= 80 && $3 < 2.0' 5BOW_chainA_Dali_PDB.txt > dali_output_5bow_filtered.txt
echo "DALI hits filtered and saved to dali_output_5bow_filtered.txt"

# === FIGURE 7b: Search AFdb using Foldseek with distinct domain architectures ===

echo "Downloading and setting up Foldseek..."
wget -nc https://mmseqs.com/foldseek/foldseek-linux-avx2.tar.gz
tar xvzf foldseek-linux-avx2.tar.gz
export PATH=$(pwd)/foldseek/bin/:$PATH

echo "Creating Foldseek database..."
foldseek databases Alphafold/UniProt up tmp_up

# Search with two query PDBs from PDB domain-architecture representatives
echo "Running Foldseek search against AFdb..."
foldseek easy-search query_structure1.pdb query_structure2.pdb up foldseek_results_combined_1_2.txt
tmp_up \
  --max-seqs 5000 -e 1e-4 --alignment-type 0 \
  --format-output target,alnlen,qlen,qcov,rmsd,taxname,taxlineage

# Filter: RMSD < 3 Å and alignment >= 30% of query length
echo "Filtering Foldseek hits for RMSD < 3 and coverage ≥ 30%..."
awk -F'\t' '($4+0 < 3) && ($3+0 >= 0.3 * $2) { print }' foldseek_results_combined_1_2.txt >
foldseek_filtered_results.txt
echo "Foldseek hits filtered and saved to foldseek_filtered_results.txt"

# === FIGURE 7c: Prepare for RMSD vs Sequence Identity plots ===

# echo "To create RMSD vs Sequence Identity plots for hits with >80% query coverage:"
# echo "Please extract RMSD (column 4) and % identity (if available) from the filtered results."
# echo "Use your preferred plotting tool (e.g., Python/matplotlib, R/ggplot2)."
# echo "This will correspond to the plots shown in Figure 2."

echo "All pipeline steps completed."
```
