# Supplementary material for: Tracing the function expansion for a primordial protein fold in the era of fold-based function prediction: β-trefoil
Source: PLoS One. 2025 Jul 3;20(7):e0320177. doi: 10.1371/journal.pone.0320177 (PMC12225799; doi:10.1371/journal.pone.0320177)
Supplement: S4 File — (PDF) [file pone.0320177.s004.pdf]

```
from Bio import AlignIO
from collections import Counter
import math
import matplotlib.pyplot as plt
import csv

# --- Parameters ---
msa_file = "All_NR_structurally_aligned.afasta"  # Replace with your filename
entropy_threshold = 2.0           # Change if needed
csv_output_file = "entropy_results.csv"
plot_output_file = "entropy_plot.png"

# --- Load Alignment ---
alignment = AlignIO.read(msa_file, "fasta")
alignment_length = alignment.get_alignment_length()

# --- Function to compute Shannon entropy per column ---
def shannon_entropy(column):
    counts = Counter(column)
    total = sum(counts.values())
    entropy = 0.0
    for aa, count in counts.items():
        if aa == '-':  # ignore gaps
            continue
        p = count / total
        entropy -= p * math.log2(p)
    return entropy

# --- Calculate entropy per position ---
entropies = []
for i in range(alignment_length):
    column = alignment[:, i]
    entropy = shannon_entropy(column)
    entropies.append(entropy)

# --- Write to CSV ---
with open(csv_output_file, "w", newline='') as csvfile:
    writer = csv.writer(csvfile)
    writer.writerow(["Position", "Entropy"])
    for i, e in enumerate(entropies, start=1):
        writer.writerow([i, e])

print(f"Entropy values saved to '{csv_output_file}'.")

# --- Plotting ---
positions = list(range(1, alignment_length + 1))
high_entropy_positions = [i for i, h in enumerate(entropies) if h >= entropy_threshold]

plt.figure(figsize=(15, 5))
plt.plot(positions, entropies, label="Shannon Entropy", color="blue")
plt.scatter(
    [positions[i] for i in high_entropy_positions],
    [entropies[i] for i in high_entropy_positions],
    color='red',
```

```
    label=f"High Entropy (≥ {entropy_threshold})"
)
plt.axhline(y=entropy_threshold, color='gray', linestyle='--', linewidth=1)
plt.xlabel("MSA Position")
plt.ylabel("Entropy")
plt.title("Shannon Entropy per MSA Position")
plt.legend()
plt.tight_layout()

plt.savefig(plot_output_file, dpi=300)
plt.close()
print(f"Plot saved as '{plot_output_file}'.")
```
